# Suicidality Among Men in Russia: A Review of Recent Epidemiological Data

**DOI:** 10.7759/cureus.22990

**Published:** 2022-03-09

**Authors:** Val Bellman, Vaishalee Namdev

**Affiliations:** 1 Psychiatry, University of Missouri, Kansas City School of Medicine, Kansas City, USA; 2 Medicine and Surgery, Mahatma Gandhi Medical College and Research Institute, Indore, IND

**Keywords:** psychiatric epidemiology, chronic alcoholism, self-harm, russia, suicide risk

## Abstract

Suicide is a phenomenon that is not related to a specific class of countries but is a problem worldwide. Many studies have attempted to explain gender differences in suicidal behaviors. Unfortunately, Russia holds the world’s top place for the number of suicides committed by its male citizens. Russia is still demonstrating unusually high death rates due to non-natural causes, and these demographic trends are concerning. We analyzed suicidality among men in Russia over the past 20 years using official data published by the Federal State Statistics Service (Rosstat) and secondary sources. We also discussed male suicide as a social problem, analyzed, and evaluated male suicidality in Russia from 2000 to 2020, and reviewed the factors influencing the prevalence of male suicides over female suicides in Russia.

Russia is still going through one of the most significant historical changes in the last 100 years. Our analysis showed discrepancies between official numbers and data published by non-government organizations in Russia. Unemployment, low socioeconomic status, underdiagnosed and/or untreated mental illness, and substance abuse are major risk factors for suicide in Russian men. Cultural influences also make suicidal behavior socially scripted in Russia.

By providing examples and analyzing data, we aspire to encourage improvements in the practice of mental wellbeing in Russia and other post-Soviet countries. The recommendations within this report are intended as a starting point for dialogue to guide effective suicide prevention in this country.

## Introduction

Suicides and self-harming behaviors are significant public health and social problems in post-Soviet Russia. Suicide is one of the leading causes of death worldwide [1], accounting for over 58,000 deaths annually in Europe [2] and 16,546 deaths in Russia in 2020 [3]. According to experts, there are 11.4 suicides per 100,000 people in the world, which equates to 804,000 suicides annually [4]. Although the suicide rates in Russia are gradually decreasing (39.1/100,000 in 2000 to 23.4/100,000 in 2010 and 11.3/100,000 in 2020 [3]), the number of suicides among men is significantly higher than among Russian females [5, 6].

The suicide rates vary greatly between Russian cities and within the country, and the difference between regions varies tenfold. The suicide rates are higher in rural communities when compared with their urban counterparts. Social deprivation, economic depression, unemployment, heavy alcohol consumption, etc. are also more prevalent in rural areas of Russia. Indigenous peoples around the country are burdened with a markedly increased suicide rate, which may be associated with a challenging social situation, inadequate family support, lower socioeconomic status, and an increased prevalence of alcohol and psychoactive substances, which also act as suicide risk factors in general [7, 8]. The suicide rates among men in Russia (26.1 per 100,000) were over three times higher than among women (6.9 per 100,000) in 2016. Committing suicide appears to be a male phenomenon over the past 20 years in post-Soviet Russia [9]. For suicide attempts, the level estimated by the World Health Organization (WHO) is 20 times higher than the suicide rate [10]; the gender gap is less pronounced.

This phenomenon, when men commit suicide more frequently than women while women are much likelier to commit suicide attempts, is known as the gender paradox of suicidal behavior [2, 6]. All Russian citizens are expected to receive medical care that meets the highest standards, regardless of their race, religion, national origin, sexual orientation, gender identity, or expression. Although the Russian healthcare system remains gender-neutral, Russian men are not considered a “risk” group and are not involved in targeted state-sponsored suicide prevention programs [11].

## Materials and methods

Data on the population and male suicide rates were taken from the official reports of Rosstat and the Ministry of Health of the Russian Federation for 2001-2020. Secondary data were obtained from international databases and published studies in Russian and English. We used descriptive statistics to summarize the information about the population being studied. This methodology helped us summarize data in the form of simple quantitative measures, such as percentages and means, or visual summaries, such as diagrams and bar charts. The literature review attempted to bring together all available evidence on a specific, clearly defined topic.

Published studies were identified through ‘pearl growing’, citation chasing, a search of databases, using the filters, and the authors’ topic knowledge. The articles were searched in MEDLINE, PubMed, EMBASE, COCHRANE, eLibrary, and CyberLeninka. A search of databases was undertaken in December 2021 using predefined keywords. Citation chasing was conducted by analyzing the references for each included study. A total of 122 potential papers were identified. We also included at least 20 Russian biomedical journals listed in databases, which were translated into English. The summary document contained the list of included and excluded articles; the inclusion status for each article was based on a review of the full-text manuscript. The inclusion criteria were articles with the target population, specific location, investigated epidemiological trends, or the comparison between two-to-three studied regions (cities, states, or districts). Exclusion criteria were unrelated, duplicated, unavailable full texts published before 2001. Data were abstracted from 60 eligible papers. Some of these sources had English-language abstracts, but other articles’ texts had to be translated. The evidence was graded for each source based on the quantity and quality of studies and potential data flaws. The quality, validity, and type of published data were considered. 

The citation management system EndNote allowed us to organize our literature databases with internet searches and have add-ons for Office programs, which made the process of literature citation convenient. However, the majority of articles in Russian could not be captured by the citation management system. Additionally, the search for article content was sometimes unavailable for search engines. The authors had to enter this information manually to ensure consistency in the referencing of studies. Some Russian sources were originally published as extensive PDF files of the entire journal issue without dividing it into separate articles and providing no descriptors, making manual, time-consuming input of information the only possibility.

## Results

Not only are men likelier to die of suicide than women between the ages of 10 and 60 years, but the suicide rate among men also grows with every decade of life, reaching a peak at 50 [12, 13]. Russian men become increasingly inclined to commit suicide before their 60th birthday, usually via firearms or strangulation. Although men aged 60, 70, and 80 die from suicide less often than men aged 40 to 59, gender differences prevail. The suicide rate among men over 60 is about 30 cases, compared to about 10 (per 100,000 people) among women of the same age [11, 12]. 

**Figure 1:**
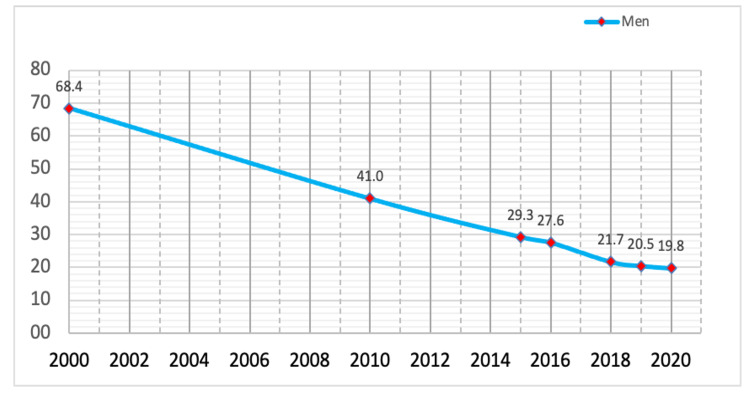
Suicide rate among men in 2000-2020 per 100,000 people

Official data illustrate that suicide rates among men have gradually decreased over the past 20 years. While in 2000 it was 68.4 cases per 100,000 people, in 2010, it was 41 cases per 100,000 people, gradually decreasing to 29.3, 27.6, 21.7, 20.5, and 19.8 cases in 2015, 2016, 2018, 2019, and 2020, respectively, per 100,000 people. Suicide mortality among women is significantly lower than among men. In 2015-2016, it was nearly four times lower than among men and amounted to 7.5 and 7.1 cases per 100,000 people, respectively, in 2015 and 2016. The suicide rate among men in 2000-2020 per 100,000 people is shown in Figure 1 [3].

**Figure 2:**
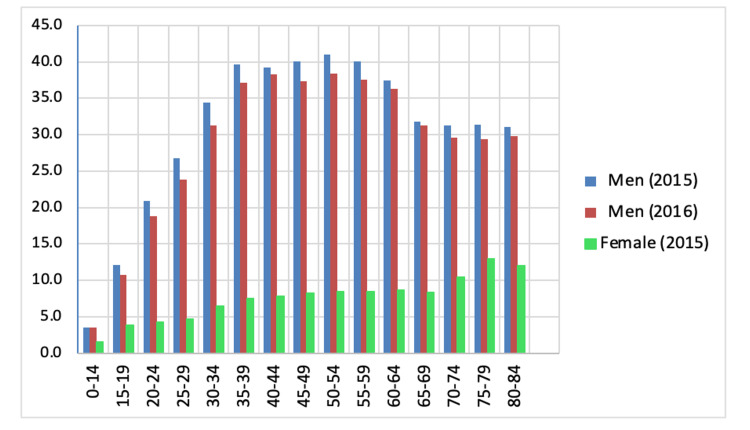
Changes in suicide mortality in 2015–2016 per 100,000 people, depending on age group

According to official data, the suicide rate among all age groups decreased. In recent years, the suicide rate among adult men has varied. Data demonstrate that the suicide rate among men increases with every decade of life, reaching a peak of 50 years. Thus, at the age of 15-19 years, the mortality rate from suicide among men was 10-12 cases in 2015-2016 per 100,000 people, at the age of 20-24 years: 18-20 cases, 25-29 years: 24-26 cases, 30-34 years: 31-35 cases, 35-39 years: 37-40 cases, and reaching a maximum in the age group of 50-54 years at 38-41 cases, then decreases gradually. Figure 2 summarizes data on male suicide mortality in 2015-2016, depending on the age per 100,000 people [12].

**Figure 3:**
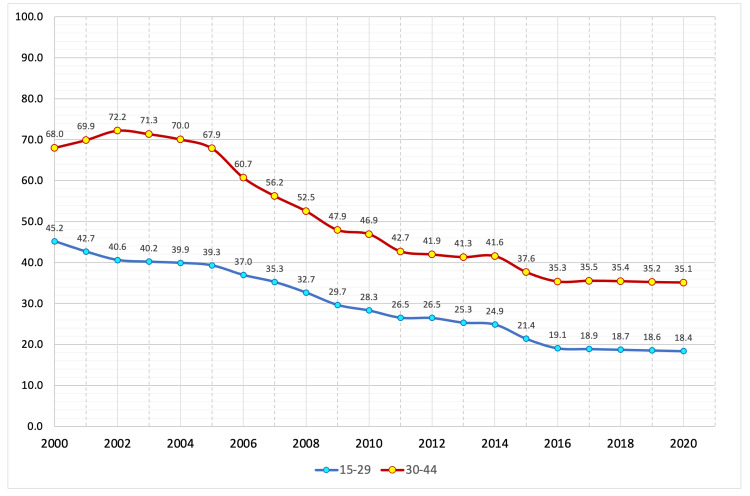
The male suicide mortality rate in the age groups of 15-29 and 30-44 years in 2000-2020 per 100,000 people

**Figure 4:**
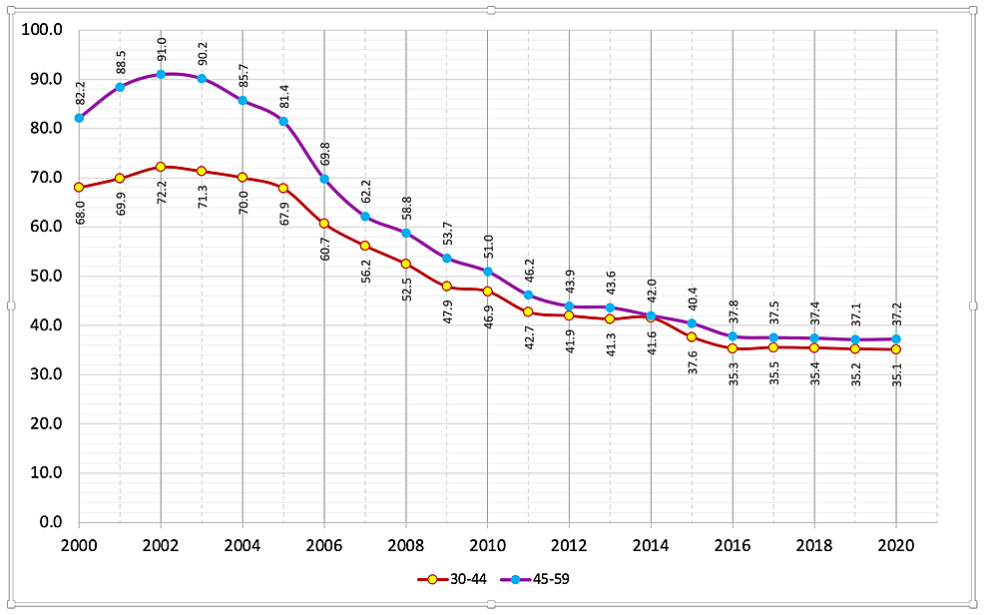
Male suicide mortality rate in the age groups 30-44 and 45-59 years in 2000-2020 per 100,000 people

**Figure 5:**
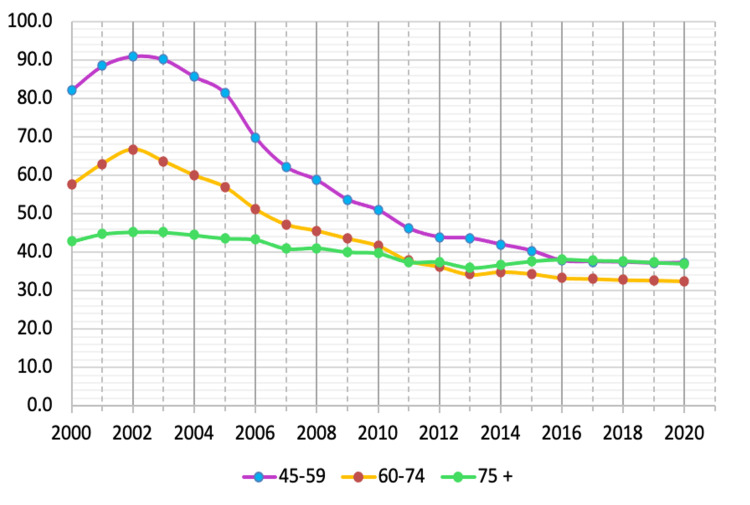
Male suicide mortality rate among the male population in the age groups 45–59, 60–74, over 75 years in 2000–2020 per 100,000 people

The data show that the suicide mortality rate among the male population in various age groups has been steadily decreasing since 2002. Between 2000-2003, all age groups of the male population demonstrated a growth in the number of suicide cases. It peaked in this period (2000-2020), except for the 15-29 age group. Between 2004 and 2010, there was the fastest decline in the suicide mortality rate among the male population in different age groups, after which the rate of decline in the mortality rate slowed, which may have been due to the financial and economic crisis in Russia (2008-2010). Figures 3-5 summarize the changes in the suicide mortality rate among men in different age groups in 2000-2020 [3].

Between 2000-2020, the male suicide rate was variable across all levels of urbanization with higher rates in nonmetropolitan/rural areas than in medium or large metropolitan clusters. Geographic disparities (specific federal districts versus Russia overall) in suicide rates might reflect suicide risk factors known to be prevalent in less urban areas, such as limited access to mental health care, social isolation, and substance abuse.

**Figure 6:**
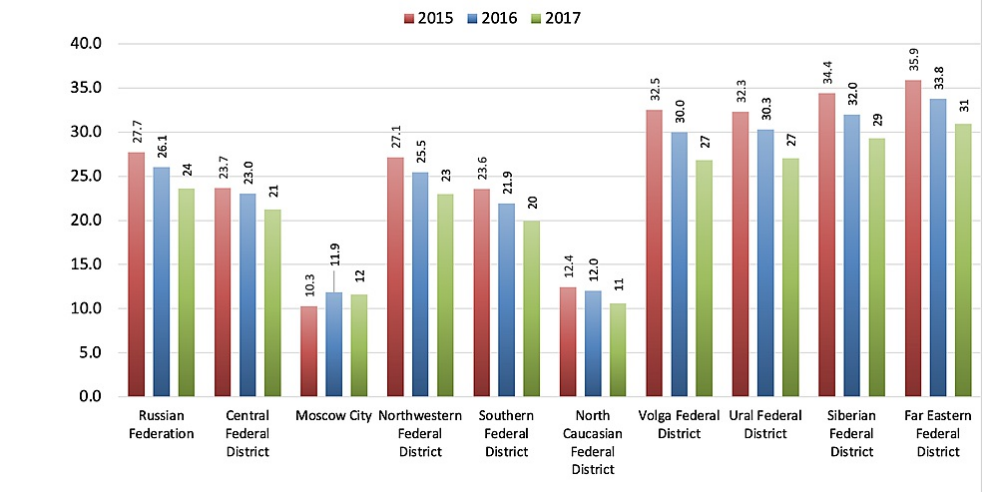
Male suicide mortality rates in various federal districts and the Russian Federation in 2015–2017

Official data show that in 2015-2017, the suicide mortality rates among the male population in the Central Federal District, the city of Moscow, and the North Caucasian Federal District were lower than the average for the Russian Federation. The lowest rates were seen in the city of Moscow and the North Caucasian Federal District. In the Northwestern Federal District, suicide mortality rates among the male population were about the same as those in the Russian Federation overall. In the Volga Federal District, Ural Federal District, Siberian Federal District, and Far Eastern Federal District, suicide mortality rates among the male population were higher than the average in Russia. Figure 6 summarizes the male suicide mortality rates in various federal districts and the Russian Federation in 2015-2017 [11, 12]. 

Interestingly, Mal et al. (2020) stated that the highest suicide mortality rates were in five Russian federal districts: Northwestern, Volga, Ural, Siberian, and Far Eastern; however, their analysis focused on suicide mortality rates in general. Additionally, the authors indicated that suicide mortality rates were significantly lower in Central, Southern, and North Caucasian Federal Districts [14].

**Figure 7:**
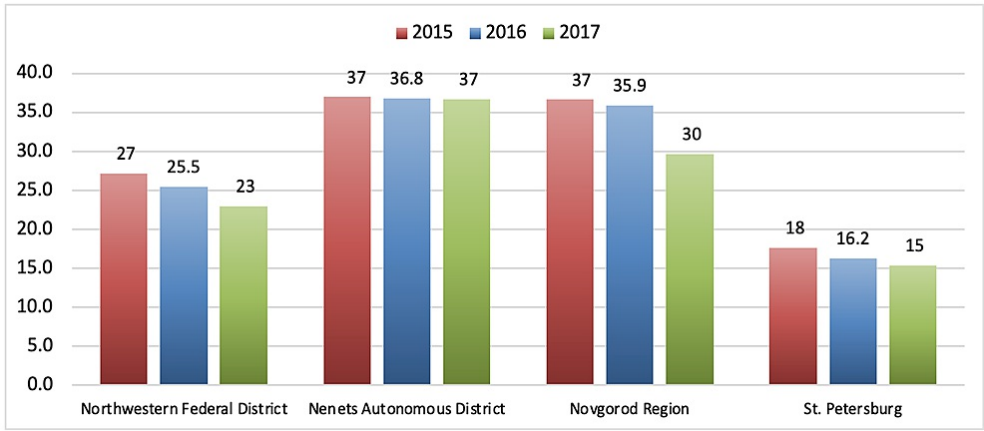
Male suicide mortality rates in various regions of the Northwestern Federal District in 2015–2017

The impact of urbanization on suicidality in Russian men and on the mental health of the general population remains underestimated [15]. The highest degree of urbanization was recorded in the Northwestern Federal District of Russia, where almost 85 percent of the inhabitants lived in city areas. The extent to which the suicide rate in urban areas is influenced by exposure to risk factors other than urbanization remains unknown due to a lack of data. The lowest male suicide mortality rates in the Northwestern Federal District are seen in the city of St. Petersburg, where these numbers are lower than the indicators for the Northwestern Federal District. Suicide mortality rates among the male population in the Northwestern Federal District decreased in 2015-2017. The most significant decrease occurred in the Novgorod region. Figure 7 shows the suicide mortality rates among the male population in various regions of the Northwestern Federal District in 2015-2017 [11, 12].

**Figure 8:**
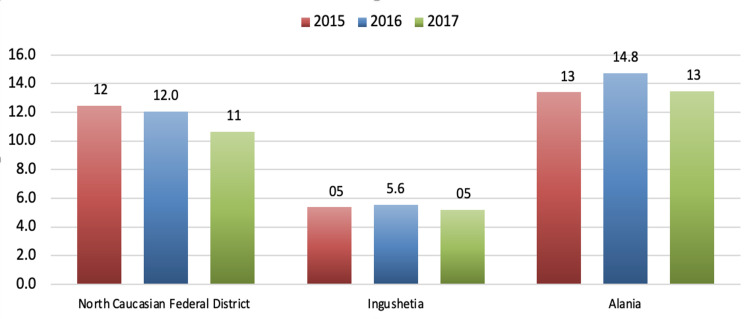
Male suicide mortality rates in various regions of the North Caucasian Federal District in 2015–2017

Interestingly, the regions located in the Northern Caucasus demonstrate significantly lower male suicide rates compared to the rest of the nation [16]. These numbers and demographic trends were noted almost 20 years ago and remain consistent with our data. The published data suggest that the highest suicide mortality rates among the male population in the North Caucasian Federal District were in the Republic of Alania, being higher than the indicators for the North Caucasian Federal District by about 15%. The lowest male suicide mortality rates were in the Republic of Ingushetia. The numbers are lower than these indicators for the whole North Caucasian Federal District by over two times. These male suicide mortality rates are the lowest of those discussed in this report. However, higher suicide rates were found among male soldiers who served in the Chechen wars and/or were actively serving in other areas of the Caucasus [17]. Figure 8 shows suicide mortality rates among the male population in various regions of the North Caucasian Federal District in 2015-2017 [11, 12].

**Figure 9:**
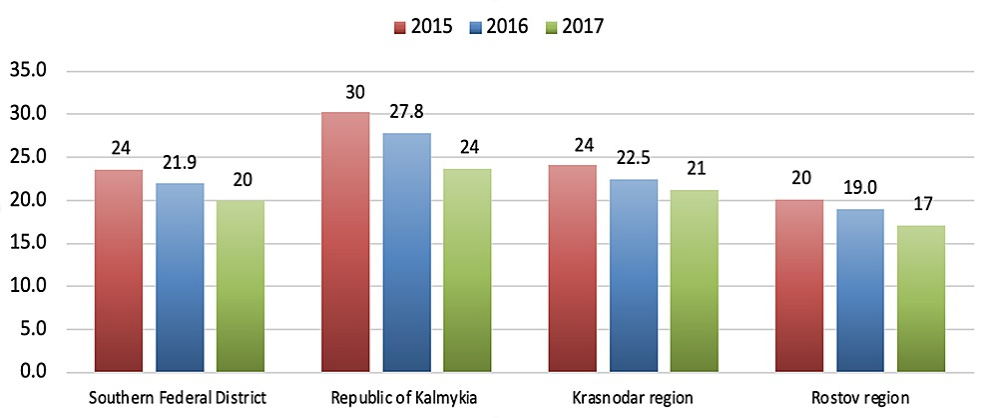
Male suicide mortality rates in various regions of the Southern Federal District in 2015–2017

Interestingly, the Russian Southern Federal District borders the republics of the North Caucasus. While some parts of that district are ethnically like the North Caucasus, the male suicide mortality rates are like other regions of Russia with a predominantly Slavic population. Data on male suicide mortality rates in various regions of the Southern Federal District from 2015-2017 showed a gradual tendency to decrease, but those numbers are still significantly higher than in the North Caucasus region. In the Republic of Kalmykia, suicide mortality rates among the male population in 2015-2017 were higher than in the Southern Federal District by about 20%. In the Rostov region, suicide mortality rates among the male population in 2015-2017 were about 15% lower than those in the Southern Federal District. Figure 9 illustrates suicide mortality rates among the male population in various regions of the Southern Federal District in 2015-2017. 

**Figure 10:**
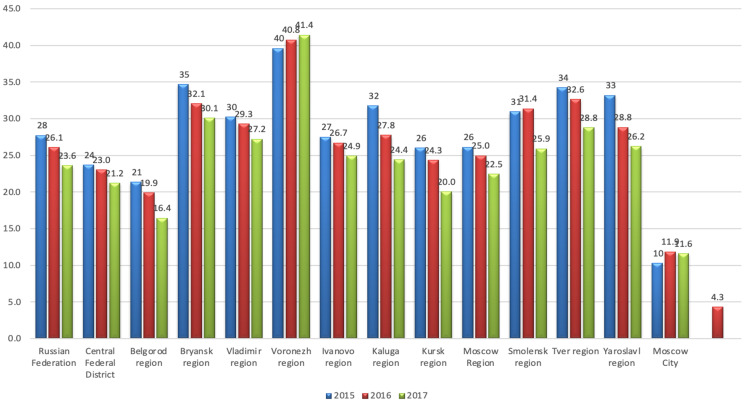
Male suicide mortality rates in various regions of the Central Federal District and the Russian Federation in 2015–2017 The red column (4.3) is the suicide mortality rate among the female population in Moscow in 2016 [11, 12]

The Central Federal District is located in the center of the European part of Russia. It is the district with the highest density of population in Russia-60.30 people per square kilometer: a high level of urbanization, as about 50% of the population lives in the Moscow region. This region has a high level of economic and social activity and a presumably better socioeconomic situation. However, male suicide mortality rates vary between cities. Suicide mortality rates among the male population in the Belgorod Region and the city of Moscow were lower than in the whole Central Federal District. In the Kursk and Moscow regions, mortality rates were about the same as in the Central Federal District, especially in 2017. In the regions of Bryansk, Vladimir, Voronezh, Ivanovo, Kaluga, Smolensk, Tver, and Yaroslavl, suicide mortality rates among the male population were higher than in the Central Federal District. In 2015-2017, nearly all regions of the Central Federal District demonstrated decreased male suicide mortality rates. The fastest rates of decline were observed in the regions of Belgorod, Kursk, Smolensk, and Tver. In the Voronezh region, there was an increase in the death rate from suicide among the male population. In Moscow in 2016, the suicide mortality rate increased among the male population compared to 2015. In 2017, this index dropped again. Males aged 55 years and older were more likely to die from suicide than any other age group for both males and females. Figure 10 shows male suicide mortality rates in various regions of the Central Federal District and the Russian Federation in 2015-2017 [11, 12].

Male suicides in the Volga Federal District showed a linear trend of decline in 2015-2017, despite the risk factors for suicide generally increasing. The most significant decrease in male suicide mortality rates among the male population was observed in the Saratov region, which initially showed an unexpected increase in male suicide rates (higher than in the Volga Federal District by about 23%) [11, 12]. Suicide mortality rates among the male population in the Ural Federal District in 2015-2017 also showed a tendency to decrease [11, 12]. 

Social marginalization and depopulation are particularly widespread in regions of the Asian part of the country. Despite the implementation of additional state-run social and demographic incentives, the impoverishment of human capital is still evident in this region. This region is far removed from Russia’s European core and financial centers but remains uncomfortably close to dynamic and powerful China. Despite the oil and gas resources of East Siberia and the Far East Federal District, its regional product amounts to just 5-6 percent of Russia’s total gross domestic product (GDP). 

**Figure 11:**
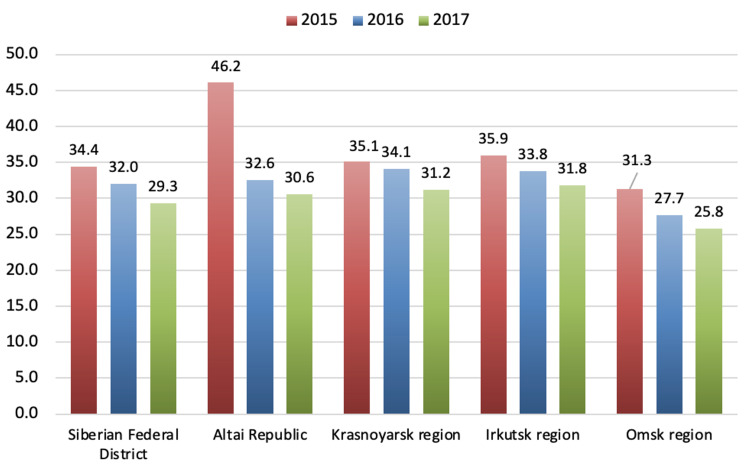
Male suicide mortality rates in various regions of the Siberian Federal District in 2015–2017

**Figure 12:**
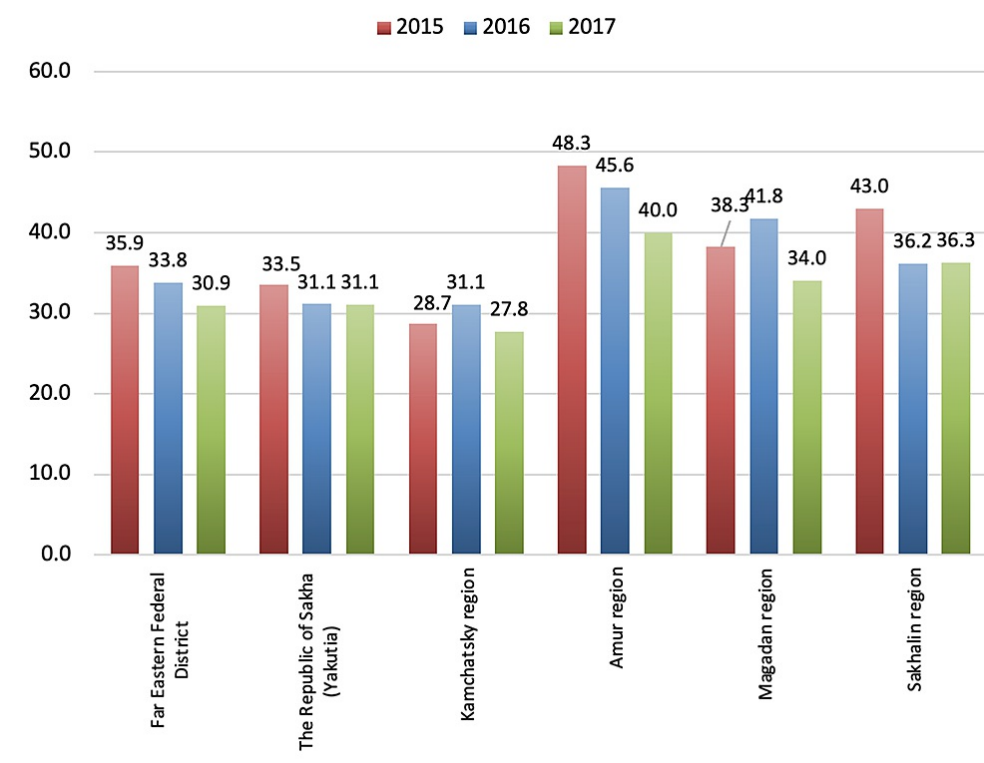
Male suicide mortality rates in various regions of the Far Eastern Federal District in 2015–2017

These two regions have long been known as underdeveloped and socially challenging. Despite these circumstances, the suicide mortality rates among the male population in the Siberian Federal District (SFD) in 2015-2017 also showed a tendency to decrease. The most significant decrease in suicide mortality rates among the male population occurred in the Altai Republic. In the Krasnoyarsk Region, the Irkutsk region, the indicators were fairly even, like the rates for the Siberian Federal District. Interestingly, in Omsk, suicide mortality rates among the male population in 2015-2017 were about 10% lower than those in the entire Siberian Federal District. The official data show that the highest male suicide mortality rates in the Far Eastern Federal District were in the Amur and Sakhalin Regions, being higher than these rates for the Far Eastern Federal District by 28% and 23%, respectively. Interestingly, the lowest male suicide mortality rates were in the Kamchatsky Territory, where these numbers were lower than the indicators for the Far Eastern Federal District by about 10-15%. Figures 11 and 12 summarize data regarding male suicide mortality rates in various regions of the SFD and the Far Eastern Federal District in 2015-2017 [11, 12]. 

## Discussion

Data accuracy issues 

According to the World Bank, Russia ranks third in the world in the suicide mortality rate, and this rate in 2019 was 25.10/100,000 per year. However, this rate is disproportionally higher for men. It is important to mention that these rates have been declining over the past 20 years. The available data highlight that the suicide mortality rate among Russian men was as high as 96.7/100,000 in 2000 and decreased to 43.60 in 2019 [18]. Interestingly, these numbers do not correlate with the data provided by Rosstat [3]. Table 1 provides additional information on this matter.

**Table 1:** Russia’s suicide rates over the past 20 years Adopted from macrotrends.com [18].

Year	Total	Male	Female
2000	53.8	96.7	16.1
2001	52.4	94.1	15.9
2002	52	93.1	16
2003	51.6	92.4	15.9
2004	50.1	89.8	15.4
2005	49.8	89.3	15.4
2006	44.8	80	14.1
2007	41.7	74.1	13.5
2008	40.7	72.5	13.2
2009	38.4	68	12.8
2010	38.1	67.5	12.6
2011	35.9	63.5	12
2012	34.4	60.9	11.6
2013	33.5	59.3	11.2
2014	33.8	60	11.3
2015	32	56.4	11
2016	31	54.4	10.7
2017	28.2	49.3	10
2018	27.1	47.2	9.7
2019	25.1	43.6	9.1

The research data published in Russia are not always transparent. For example, the “event of undetermined intent” has shown exponential growth since 2014 and has exceeded suicide mortality rates [19]. The researchers believe that this subcategory includes “latent homicides and suicides,” while actual suicide mortality rates remain unclear. Local coding and data recording standards vary significantly and can negatively affect the transparency of the data. Specifically, many suicides are frequently listed within the “external causes of morbidity and mortality” subcategory [19]. The ICD-10 classification category includes multiple “environmental events and circumstances as the cause of injury, and other adverse effects,” where potential suicides can be included without any further systematization. “Latent suicides” include falls from heights, poisoning, and hanging with unspecified intent. They account for a significant proportion of suicide mortality. Since they are counted as events of undetermined intent, statistics show a sharp drop in suicide mortality rates, which has a linear trend [20]. This approach serves as a perfect example of data distortion practices. Moreover, there is no distinct updated information regarding suicides committed in Chechnya and in other North Caucasus republics. Yumaguzin (2019) indicated that suicide rates are significantly underestimated, while ill-defined causes of death are used to misinterpret data related to suicide and self-harming behaviors [19].

According to Verbitskaya [21], 80% of publications in Russian have methodological issues or unacceptable research designs. Based on our analysis, many studies conducted or published in Russia have methodological flaws (e.g., incomparable populations, lack of standards, internationally approved scales, and different designs). An analysis of the literature published in Russian showed that many journals have no specific or evidence-based standards for the description and presentation of research results. Although these issues are not directly related to our assessment of men’s suicide rates, it is important to mention these flaws to facilitate positive changes in data reporting. No matter how much the data vary, male suicide mortality rates remain exceptionally high. 

Socioeconomic environment

Many experts agree that male suicide mortality rates are a consequence of social, economic, psychological, and demographic issues. Some of Russia’s cultural norms can be attributed to the nation’s tumultuous history, such as that of the former Soviet Union. With the fall of communism, the nation experienced social and economic hardships that adversely affected many Russians’ mental health. Some theorize that such monumental societal changes during that time have had long-term effects, persisting until the present day. However, there has been a downward trend in suicide rates over the last two decades because the nation has improved on many socioeconomic indicators [22]. The number of suicides correlates with social changes, such as resettlement, assimilation, and the destruction of the conventional social structure. 

Financial struggles can be attributed to increased suicidality in men. The three main economic indicators, which are GDP, unemployment rate, and consumer price index, are associated with suicidal ideas, suicide attempts, and suicides [23]. In the economic crises of the 1990s, unemployment and a decrease in personal income were directly correlated with growing suicide rates, especially among men [24]. Another study evaluated how certain socioeconomic factors influenced suicide patterns within Russia. The findings demonstrated a significant decline in the male suicide rate with the country’s improvement in economic indicators (e.g., income per capita, GRP growth rate, etc.). The study also evaluated the effects of marriage and divorce on suicide rates among men. Marriage has negative effects on suicide rates, while divorce has positive effects on suicide rates [25]. Russian men are more prone to relocate and tend to move to large cities to obtain employment and work on a shift basis. These difficulties have also led to the insufficient development of institutions expected to address these social issues [26]. 

Geographical aspects

People living in rural areas of Russia are at a greater risk of suicide than those living in urban areas or big cities. The strength of the connection between intoxication and suicide also depends on the geographical region in Russia. Specifically, the data show that rates increase from the south and west to the north and east of the country [24]. 

Not only are suicide rates significantly lower in the Northern Caucasus, but other factors also make it important to consider other psychosocial factors. For example, a higher proportion of Muslims in these regions results in a different cultural context in the Northern Caucasus than in the rest of Russia, plus religious differences and Islamic scriptures against suicide. Furthermore, the intersection of these cultural factors with social institutions means that several of the measures included here as controls are confounded with a location in this area.

Average alcohol consumption in central Russia is high with a relatively large proportion of unrecorded consumption ranging from almost zero to 21 liters [27]. The rates of heavy alcohol consumption (more than 40 g of pure alcohol per day) among men were the lowest in Kabardino-Balkaria and Karachay-Cherkessia (2.3 L of ethanol per adult/year) and the highest in Magadan region (24.3 L per adult/year) [28]. Alcohol consumption is lower in these regions, and wine products are more often consumed here than in the rest of the country, meaning that the preference for vodka is not as strong as elsewhere in Russia.

Cultural aspects

The Russian mentality is characterized by a man destined to serve the motherland, the army, and his family. Russian culture is rooted in rigid gender roles, and these norms are present even at the institutional level. In The ABC for Men, the author determined that Russia has over a dozen laws that discriminate against men. For example, Russian law supports the idea of motherhood among women, yet no laws exist that support fatherhood. Although there is no concept of "single father" in Russian law, the number of families consisting of single fathers with children is slowly growing in Russia (1.18% in 2002 vs. 1.27% in 2010. According to Russian law, these men are eligible for the same benefits as single mothers [29, 30, 31]. Russian legislators have attempted to pass several similar bills that, although unsuccessful, highlight the inequities between males and females.

Along these lines, men experience different expectations in terms of occupation. Women are not allowed to work certain jobs that are considered difficult or dangerous. Likewise, these occupations consist solely of male employees, allowing men easier access to suicide modalities at hazardous places of work. Such methods, such as pesticides or firearms, are more lethal. Not only this, but a man’s age of retirement is a full five years later than that of a woman [31]. These policies indicate Russian cultural pressures, which may adversely affect men’s mental health and suicide rates. Finally, 40-50 percent of all marriages in Russia will end in divorce or separation. High divorce rates may also contribute to the likelihood of higher suicide rates in this country [32].

Child and adolescent suicidality in Russia

Across all post-Soviet countries, Russia has one of the highest rates of child and adolescent suicide [33]. Parental neglect, such as physical, sexual, or emotional abuse in childhood (PSEA), is very common in Russian families. The link between PSEA and the risk of suicide throughout life has been confirmed by published research data [34]. 

According to multiple reports, Russia has often outstripped Europe when it comes to teen suicide rates [35]. The adolescent suicide rates (specifically between ages 15-34) have steadily increased since 1996, more so than the older age groups. Suicide among young Russian males is four times more common than among young females (32.8 per 100,000 people versus 7.6 in 2004), and it occurs among ever-younger males, some in their early teens [36]. Although younger groups have had consistently lower suicide rates than middle-aged and older adults, young Russian men have attempted suicide almost twice as often as female youth since 1989. According to reports, almost 4,000 teen suicide attempts were registered in Russia annually, and as many as 1,500 of them resulted in death. In 2016, an ominous report by journalist Galina Mursaliyeva in the Russian newspaper Novaya Gazeta surfaced, which brought to light the presence of online “death groups” on the social media platform vk.com, which influenced countless teenagers to commit suicide worldwide [37], the biggest proportion of which were Russian teenagers.

In turn, the administrations’ knee-jerk reactions to increasing internet censorship did little to address the situation. There was a 14% spike in emergency room trips for potential suicides by children and adolescents in 2018 compared to 2017 (692 in 2017 versus 788 in 2018), according to findings reported by state officials [38]. Local media reports estimated that adolescent suicide rates remained relatively unchanged in 2018-2019. Interestingly, local experts noted that increasingly more Russian teenagers wanted to participate in or “supervise” online suicide games in 2020-2021 [39].

The underlying conditions that deem these children more susceptible to suicidal ideations are social isolation, a dysfunctional family system (e.g. families with interpersonal conflicts, misbehavior, child abuse or neglect), increased social isolation due to stigma surrounding mental health, an inability to relate to the opposite sex, and intolerance toward LGBTQ+ youth [40, 41]. Additionally, decreased attention by caregivers to a child’s emotional needs has been the norm for a long time.

Multiple support groups, such as Your Territory and Deti 404, have since emerged on Vk.com to give teenagers a platform to express their frustrations with a skilled support network that provides counseling and mental health support [40].

Mental health and stigma

Studies of the relationship between psychopathology, substance abuse, and suicide consistently indicate that around 70% of people who die from suicide suffer from an identifiable mental disorder before death. Episodes of major depression associated with a major depressive disorder or bipolar disorder account for at least half of suicide cases [42]. The prevalence of affective disorders in Russia ranges from 30-40%. The majority of cases remain underdiagnosed and undertreated [43]. Among suicides, there are usually many factors that can increase underlying risks or interact with depression and increase suicide risk, such as alcohol- and drug-related disorders, which are more common in men [44].

In almost all regions across the country, men consistently live shorter lives than women. Especially among middle-aged Russian men, high alcohol consumption and ongoing mental health problems contributed to gender differences in all-cause mortality [45]. 

In Russia, there is a stigma associated with mental health and consequent suicide. Many Russians consider mental health disorders to be self-inflicted and do not believe in treatment. This stigma can extend to a suicidal individual’s friends, family, and mental health professionals. 

Binge drinking is commonplace among Slavic nations, with Russia being one of them. Suicides among men in Russia are specifically associated with high rates of alcoholism. Russia’s cultural pressures also affect the physical health of the country’s men. Men are discouraged from coping with life stressors in healthy ways, and many men turn to drinking or smoking to cope [31]. Data have shown that many Russian men drink alcohol to cope with stress, unemployment, depression - in situations in which they would otherwise have difficulty coping. High levels of alcoholism in Russia existed before the collapse of the Soviet Union. However, a sharp rise began in the early 1990s and has risen to one of the highest worldwide. Local officials have estimated that alcohol consumption is up to 15 liters per person per year, while consumption in the European Union and the United States is between 7 and 10 liters [31].

Vodka accounts for roughly 75% of the nation’s alcohol consumption, and approximately one-third of Russian men report binge drinking vodka at least once monthly [46]. While inebriated, individuals are more susceptible to existing mental health issues and maybe likelier to act on suicidal thoughts. It was shown that life expectancy decreased by 12% between 1990 and 1994, which was directly related to alcohol mortality [24]. Researchers estimate that 61% of male suicides in Russia involve alcohol, compared to 22% of deaths worldwide that involve alcohol [47].

Future trends 

Russia is witnessing extremely high male suicide rates. As the high suicide rate among Russian males is multifaceted, it can be difficult to develop effective solutions. Current thinking suggests that access to mental health services can lessen suicide rates. Considering all the difficulties, the transition of primarily descriptive results to specialized suicide prevention programs among men turned out to be a challenging task that requires complex medical and social approaches [48-50].

In the last two decades, the Russian Federation has introduced many measures that have yielded tangible results. In the early 2000s, the state became fully involved in the control of the alcohol market [46]. In 2006, Russia implemented an alcohol policy to control the alcohol market and contain alcohol-related poisonings. President Putin implemented the law in January 2006, which regulated the volume and quality of alcohol products. The patterns thereafter revealed important learnings as to how alcohol consumption affects suicide rates. One study determined that the 2006 policy yielded a 9% decrease in male suicide mortality. This translates into 40, 000 male lives saved yearly from suicide by restricting alcohol [24]. 

The WHO published data that, in 2003, both alcohol-related mortality and the amount of alcohol consumed per year decreased significantly [51]. In this way, the mortality of men has decreased by as much as 40%, while men’s life expectancy has increased from 57 to 68 years over the past 15 years [51]. In the early stages of the COVID-19 crisis, local experts suggested that the pandemic might lead to an increase in suicide among Russians. Official data released by Rosstat suggested that for the entire year 2020, the standardized mortality ratio due to suicides dropped by 4.1%. However, WHO experts concluded that suicide mortality in Russia is worse than officially reported. According to their report, “Suicide Worldwide in 2019: Global Health Estimates,” the suicide rates (per 100 000) were 25.1 (crude suicide rate) and 21.6 (age-standardized suicide rate), or at least twice as high as the official data [52]. Given these discrepancies in the data, it is almost impossible to predict future tendencies in men’s suicide mortality. Algorithms used to estimate suicide mortality in men are no longer valid since the data are often inaccurate.

Several effective suicide prevention programs have been implemented in Russia. For example, school- and college-based suicide prevention programs [53-55] have proven effective in reducing the number of suicide attempts among students. Programs aimed at meeting the needs of elderly people from high-risk groups were less effective due to the questionable design of those interventions [56], none of which have been implemented since 2019. 

Laws that prevent access to a particular method, be they stricter firearm control laws, restriction of access and use of blister packs of pills, lockable pesticide boxes, or bridge barriers (often in combination with a crisis intervention telephone hotline), may affect the suicide rate, even if some adjustments to those methods may occur over time [57].

While Russia, unlike the United States, does not have anything like the Second Amendment in its Constitution, it does provide its citizens with the constitutional right to self-defense. Additionally, background checks before the ownership of guns are more rigorous and consider an individual’s medical and psychological history [58]. Despite stricter laws, certain individuals could easily bypass background checks either via corrupt measures or obtain firearms via illegal channels, which is a huge market. This problem was brought to the fore, especially after the mass shooting incident in the Russian city of Kazan in May 2021, when a 19-year-old went on a shooting spree, killing nine people and injuring 23. The authorities quickly passed stricter gun control laws, which included more stringent background checks and control over illegal gun trafficking [59].

The country also saw a spate of physician deaths during the COVID-19 pandemic, in which two healthcare workers died, and one suffered serious injuries due to falling from a building. While the cause of death is still a matter of speculation, it brought into light a system underequipped to deal with the pandemic due to a short supply of equipment and manpower. Reports also highlight the apathy of the hospital administration in dealing with the sudden spike of COVID-19 cases and caring for healthcare workers, many of whom worked tirelessly even after becoming symptomatic [60].

## Conclusions

Although the suicide statistics in Russia are profound, the suicide rate may be even higher than what has been reported. One of the biggest drivers of male suicidality in Russia is the country’s cultural norms. Russia remains very rooted in tradition, and within this tradition lies unique societal pressures. Cultural and psychosocial aspects of the Russian male experience, such as gender norms, low quality of life, and alcohol consumption, are likely key contributors to the country’s high suicide rates.

Our analysis of official reports and secondary sources in Russia also confirmed that there are too many publications of poor-quality study design and statistical analysis. Finally, continuous improvement of public health policy and fundamental and translational research can contribute to reducing the future suicide rate among the male population in Russia.
